# The Diagnostic Value of ^**18**^F-FDG PET/CT in Association with Serum Tumor Marker Assays in Breast Cancer Recurrence and Metastasis

**DOI:** 10.1155/2015/489021

**Published:** 2015-03-24

**Authors:** Ying Dong, Haifeng Hou, Chunyan Wang, Jing Li, Qiong Yao, Said Amer, Mei Tian

**Affiliations:** ^1^Department of Oncology, The Second Affiliated Hospital of Zhejiang University School of Medicine, Hangzhou 310009, China; ^2^Department of Nuclear Medicine, The Second Affiliated Hospital of Zhejiang University School of Medicine, Hangzhou 310009, China; ^3^Zhejiang University Medical PET Center, Hangzhou 310009, China; ^4^Institute of Nuclear Medicine and Molecular Imaging, Zhejiang University, Hangzhou 310009, China; ^5^Key Laboratory of Medical Molecular Imaging of Zhejiang Province, Hangzhou 310009, China; ^6^Beijing 307 Hospital, Beijing 100071, China; ^7^Department of Zoology, Faculty of Science, Kafr El Sheikh University, Kafr El Sheikh 33516, Egypt

## Abstract

*Background*. After initial treatment of breast cancer (BC), monitoring locoregional recurrence and distant metastases is a great clinical challenge. *Objective*. To evaluate the efficacy of PET/CT in association with serum tumor makers in BC follow-up. *Methods*. Twenty-six women with a history of modified radical mastectomy were evaluated by ^18^F-FDG PET/CT. The results of PET/CT were compared with those of conventional imaging techniques (CITs) (including mammography, chest radiography, CT, MRI, ultrasound, and bone scintigraphy). Serum tumor markers of CEA, CA 125, and CA 15-3 in the BC patients were also analyzed in association with the results of PET/CT. *Results*. Compared with CITs, PET/CT was more sensitive to detect the malignant foci and had better patient-based sensitivity and specificity. The mean CA 15-3 serum level was significantly higher in the confirmed positive patients of PET/CT results than in the confirmed negative ones, while there were no significant differences in the serum levels of CEA and CA 125 of both groups. *Conclusion*. PET/CT is a highly efficient tool for BC follow-up compared with CITs. The high serum levels of CA 15-3 in confirmed positive PET/CT patients indicated the clinical value of CA 15-3 in BC follow-up.

## 1. Introduction

Breast cancer (BC) is recognized as the most leading cause of death in women worldwide, with progressively increased incidence over the last few decades [1]. Approximately 30% of patients diagnosed with BC are at the risk of developing locoregional recurrence or metastasis to distant organs [2].

After initial treatment of BC, follow-up based on clinical examination and conventional imaging techniques (CITs) is common practice. However, localization of metastases or recurrences remains a serious challenge, requiring an extensive diagnostic workup. Mammography is of high value in the follow-up of BC and is recommended to diagnose or exclude local recurrence [3, 4], but it encounters obvious challenge to detect the remote metastases. The use and efficacy of other modalities such as chest radiography, computed tomography (CT), magnetic resonance imaging (MRI), ultrasound, or bone scintigraphy (BS) remain controversial [5–8].

Previous studies have suggested that fluorine-18 fluorodeoxyglucose-positron emission tomography (FDG-PET) might improve the sensitivity of detection recurrence of BC compared to CITs [9, 10]. Moreover, FDG-positron emission tomography/computed tomography (PET/CT) generates invaluable data on the functional activity of the recurrence sites and a general picture based on whole-body acquisition, with a high signal-to-noise ratio [11].

Blood levels of tumor markers (TMs) seem to be correlated with the tumor mass and considered as useful tool in both diagnosis and follow-up of certain cancers. In recent decades, TMs such as carcinoembryonic antigen (CEA) and cancer-associated antigen 15-3 (CA 15-3) have been used as reliable evidence of distant metastasis of BC [12, 13]. Cancer-associated antigen 125 (CA 125) is commonly used in ovarian cancer [14] and showed elevated levels in ~84% of metastatic breast patients [15, 16]. And the PET/CT has also been recognized as valuable modality for the follow-up of BC patients with elevated levels of TMs [11, 17].

The aim of this study was to evaluate the efficacy of using ^18^F-FDG PET/CT along with serum TMs (CEA, CA 125, and CA 15-3) in monitoring the recurrence and metastasis of BC in order to optimize their utility in clinical practice.

## 2. Patients and Methods

### 2.1. Patients

The retrospective study was done on a total of 26 female patients (aged 33–84 years; mean ± SD, 54.9 ± 12.1 years) with a history of modified radical mastectomy (MRM), at the Second Affiliated Hospital of Zhejiang University School of Medicine, Hangzhou, China. The patients were diagnosed as suspicion of recurrence and referred to for whole-body ^18^F-FDG PET/CT scanning at the PET Center from July 2013 to January 2014. The median time interval from initial MRM to ^18^F-FDG PET/CT was 6.3 (1–34 years). The current study was performed under informed written consent of all patients who contributed in the study. Particular attention was paid to maintain good wellbeing of all patients.

### 2.2. ^18^F-FDG PET/CT Scanning and Image Analysis

^18^F-FDG was produced by the cyclotron (Sumitomo CYPRIS HM-12S) and PET/CT was performed using the PET/CT system (Siemens Biograph mCT). All patients were instructed to fast for at least 6 hours before imaging. At the time of the tracer injection, patients should have had a blood glucose level of less than 140 mg/dL. Before and after injection, patients were kept lying comfortably in a quiet, dimly lit room. Image acquisition was started 1 hour ± 10 minutes after intravenous administration of FDG (7.4 MBq/kg body weight).

The PET/CT images were assessed by 2 experienced physicians in consensus. In the case of divergent evaluation, a third nuclear medicine specialist served as a referee. The PET images were inspected visually for regions of focally increased glucose uptake and quantitatively by detecting the maximum standardized uptake value. In equivocal findings, a standardized uptake value (SUV) of greater than 2 was considered as malignant, except the liver, where SUV higher than 2 in the difference of the focus and the surrounding normal tissue was considered as malignant [17]. Focally increased uptake located in the lung was considered as malignant in all cases. To be classified as confirmed positive, a recurrence confirmation was required, based on CITs, pathology, or clinical follow-up. To be classified as negative or false positive, a minimum of 6 months of follow-up was required, with negative CITs and/or repeated PET/CT imaging and clinical examination.

### 2.3. CITs Imaging

CT, chest radiography, and ultrasound were performed for all patients, mammography in 12 patients, MRI in 4 patients, and BS in 12 patients. All results of CITs in the time period of 3 months before and after PET/CT were collected for further analysis.

### 2.4. Tumor Markers

The protocols used to measure CEA, CA 125, and CA 15-3 concentrations were standardized for all patients. The serum CEA, CA 125, and CA 15-3 concentrations were determined by the electrochemiluminescence method. The serum CEA, CA 125, and CA 15-3 of 5.0 ng/mL, 35 U/mL and 30 U/mL, respectively, were adopted as the upper limits of normal. In combined use of the three TMs, we classified patients into “TM positive” if at least one of these markers exceeded its cut-off value.

### 2.5. Statistical Analysis

Generated data were analyzed statistically using SPSS software, version 16.0 (SPSS Inc., Chicago, IL). Results were expressed as the mean ± SD. McNemar test was used to analyze findings of PET/CT and CITs. The serum levels of CEA, CA 125, and CA 15-3 in the BC patients with confirmed positive PET/CT results were compared to the confirmed negative ones using the Mann-Whitney test. Values of *P* < 0.05 were considered significant.

## 3. Results

In the present study, CITs detected a total of 47 suspicious lesions in 18 out of 26 patients, with 28 lesions being proved to be malignant. The CITs revealed 2 local recurrences in 2 patients and detected brain metastases in one case. Four cases were identified to have 7 suspicious lung metastases, and 6 lesions were proved to be malignant in 3 patients by follow-up. In addition, 3 lymphatic metastases were detected in one case but only one lymph node metastasis located in the axilla was proved after biopsy. A total of 34 suspicious bone metastases were detected in 12 women and 18 of these lesions were proved to be malignant by clinical follow-up and rechecked by CITs. Of these, 6 were located in the ribs, 4 in the pelvis, 5 in the spine, 2 in the extremities, and 1 in the scapula.

PET/CT detected a total of 135 suspicious malignant foci in 21 out of 26 patients, with 122 lesions being proved to be malignant in follow-up or recheck by CITs. Of these, 4 were revealed to be local recurrences in 2 cases. A total of 17 foci were lymphatic metastases in 5 cases, with 3 being detected in the axilla, 13 in the mediastinum, and one in inguina. The PET/CT revealed 91 bone metastases in 9 patients, of which 24 were in the ribs, 27 in the pelvis, 26 in the spine, 2 in the scapula, and 12 in the extremities. Also, one metastasis was detected in the liver of one patient, one metastasis in brain of another patient, and 8 pulmonary metastases in 4 patients.

Collected results (Table 1) indicate that PET/CT detected significantly (*P* < 0.01) more malignant foci, at all anatomical niches but the brain, compared to CITs. The PET/CT was superior in detecting recurrence (4 versus 2) as well as metastases in lymph node (17 versus 1), bone (91 versus 18), and lung (8 versus 6). A liver metastasis was only detectable by PET/CT.

The patient-based sensitivity and specificity of PET/CT were 95.0% and 71.43% compared to 78.95% and 57.14% for CITs. The negative and positive predictive values were 100.0% and 90.48% for PET/CT versus 50.0% and 83.33% for CITs, respectively.

CEA, CA 125, and CA 15-3 levels were determined in the sera of 20 out of 26 patients. A total of 6 BC patients were classified as TM positive. Serum levels of CEA in 2 patients, CA 125 in 1 patients, and CA 15-3 in 2 patients were above normal values. Only one patient had higher serum CEA, CA 125, and CA 15-3 concentrations than normal values simultaneously. According to the PET/CT results of these 20 patients, 13 were confirmed positive and 7 were confirmed negative. There was no significant difference in CEA serum levels of confirmed positive compared to confirmed negative PET/CT patients (7.90 ± 20.34 versus 2.57 ± 2.86 ng/mL; *P* = 0.35), as well as CA 125 (15.77 ± 26.69 versus 7.41 ± 2.26 U/mL; *P* = 0.96). But the mean CA 15-3 serum level was significantly higher in the positive compared to negative ones (18.42 ± 12.13 versus 11.8 ± 9.64 U/mL; *P* = 0.04).

## 4. Discussion

Early detection and adequate localization of recurrence are essential for guiding optimal therapy for BC patients [18]. Recent studies have shown the relevance of ^18^F-FDG PET/CT in detecting distant metastasis in patients with clinical suspicion of recurrence [8–11] and in patients with confirmed locoregional recurrence [19]. Furthermore, PET/CT has been reported to have a major impact on managing BC patients with elevated TMs levels [11, 20], subsequently, leading to change of management protocols of 36%–54% of patients. The combined anatomical-molecular PET/CT imaging technique has been shown to improve significantly the specificity of FDG-PET and CT [21, 22].

**Figure 1:**
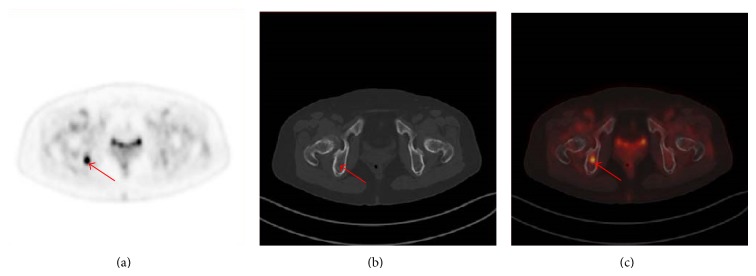
The PET image (c) demonstrates focal increased FDG uptake in right body of ischium (red arrow), which is considered as malignant. In the CT image (b), no abnormality is seen. The PET/CT (a) is considered as suspicious malignant because of the PET findings. Bone metastatic involvement of the right body of ischium was proved by follow-up.

Results of the present study confirmed that ^18^F-FDG PET/CT was an accurate technique for the appropriate detection of BC metastasis and/or recurrence compared to CITs. Compared with CITs, PET/CT detected more malignant foci overall (*P* < 0.01) (Figure 1). In addition, PET/CT was superior on CITs in terms of patient-based sensitivity, specificity, positive predictive value, and absolute negative predictive value in detecting metastases and/or recurrence. There occurred only 11 false-positive findings, 1 in the lung and 4 in the lymph nodes because of inflammatory disorder. The other 3 false-positive foci were in ribs for old fractures.

**Figure 2:**
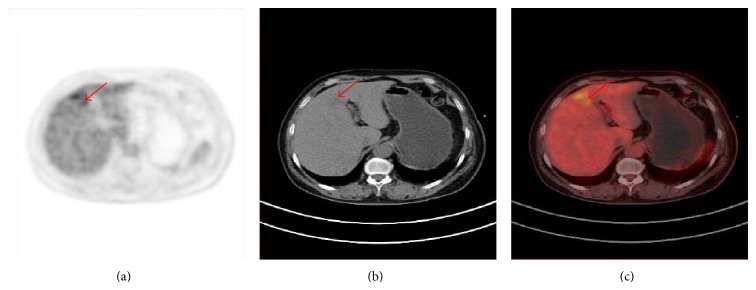
PET, CT and PET/CT of a 59 year old patient, referred for restaging because of elevated CA 15-3 level 12 years after surgically resected breast carcinoma of the left breast. The PET demonstrates a focal increased FDG uptake in the liver (red arrow), which is considered as malignant. The CT shows a slightly low dense area in the left lobe of liver. The PET/CT is considered as suspicious because of the PET findings. Metastatic involvement of the liver was proved by follow-up.

For many malignancies, the potential uses of serum TMs include aiding early diagnosis, determining prognosis, prospectively predicting response, or resistance to specific therapies, surveillance after primary surgery, and monitoring therapy in patients with advanced disease. However, in BC, the role of serum TMs is less well established [12, 23]. Recently, in the study of Yerushalmi et al. [16], TM elevation of CA 15-3, CEA, and/or CA 125 was documented in the majority of patients with metastatic BC, with CA 15-3 occurring most commonly. Zissimopoulos et al. [8] reported CA 15-3 showed a sensitivity of 67%, specificity of 74%, positive predictive value of 63%, and negative predictive value of 77% in revealing bone metastasis. Further, in the asymptomatic BC patients with elevated TMs (CA 15-3 or CEA), ^18^FDG-PET/CT imaging is an efficient technique to detect BC recurrence [11]. In our present study, 30.77% (4/13) patients of confirmed positive PET/CT results showed rising TMs (CEA, CA 125, or CA 15-3). There were also 2 patients with confirmed negative PET/CT results to show increased CEA or CA 15-3. Although no significant difference in the CEA and CA 125 serum level between confirmed positive and confirmed negative PET/CT groups was found, CA 15-3 serum level was significantly higher in the confirmed positive ones (Figure 2). This finding was in consistence with previous study [11], which reported highly increased CA 15-3 serum level was more frequently observed in cases of multiple lesions.

The number of patients involved in the present study is relatively small, which might limit the ability to generalize the generated results. Further study on a larger population is required before drawing a more definitive conclusion. For the calculation of sensitivity and specificity, a patient-based approach was used instead of lesion-based. In general, treatment decisions are generally made based on the presence of recurrent or metastatic disease, rather than on the number of lesions involved. Consequently, it is clinically more relevant to consider the patient-based data rather than the lesion-based analyses.

## 5. Conclusion

The findings of the present study indicate that PET/CT might be advantageous in the follow-up of patients with BC compared to CITs, providing a sensitive tool for detecting metastases and locally recurrent disease. The higher CA 15-3 serum level found in the confirmed positive PET/CT patients than the confirmed negative ones indicated the increased likelihood of BC recurrence and metastasis and the clinical value of CA 15-3 in BC follow-up.

## Figures and Tables

**Table 1:** Suspected and confirmed malignant foci of CITs and PET/CT.

	Malignant lesions detected by		Malignant lesions detected by		
Locations	CITs		PET/CT		Total confirmed malignancy^*^
	Suspected	Confirmed	Suspected	Confirmed	
Local recurrence	2	2	5	4	4
Lung	7	6	9	8	8
Liver	0	0	1	1	1
Brain	1	1	1	1	1
Lymph nodes	3	1	22	17	18
Mediastinal	0	0	17	13	14
Axilla	3	1	4	3	3
Others	0	0	1	1	1
Bone	34	18	97	91	101
Ribs	8	5	27	24	28
Pelvis	5	5	27	27	30
Spine	18	5	29	26	29
Extremities	2	2	12	12	12
Scapula	1	1	2	2	2

^*^Confirmation of positive cases was done based on recurrence of cancer as indicated by CITs, pathology, and/or clinical follow-up. Confirmation of negative cases was done based on absence of abnormality as indicated by CITs, repeated PET/CT imaging as well as clinical follow-up for a period of 6 months.
